# Supplementary material for: Vinorelbine Augments Radiotherapy in Hepatocellular Carcinoma
Source: Cancers (Basel). 2020 Apr 3;12(4):872. doi: 10.3390/cancers12040872 (PMC7226273; doi:10.3390/cancers12040872)
Supplement: Supplementary file 1 [file cancers-12-00872-s001.zip › cancers-738165 supplementary final/cancers-738165 supplementary final.pdf]

cancers

MDPI

Vinorelbine Augments Radiotherapy in Hepatocellular Carcinoma

Kheng Wei Yeoh, Aldo Prawira, Muhammad Zafrie Bin Saad, Kok Ming Lee, Eric Ming Hon Lee, Gee Keng Low, Mohamed Hakim Bin Mohd Nasir, Jun Hao Phua, Wendy Wan Li Chow, Iris Jiu Hia Lim, Yusnita Binte Omar, Rebecca Zhi Wen Ho, Thi Bich Uyen Le, Thanh Chung Vu, Khee Chee Soo and Hung Huynh

Supplementary Materials:

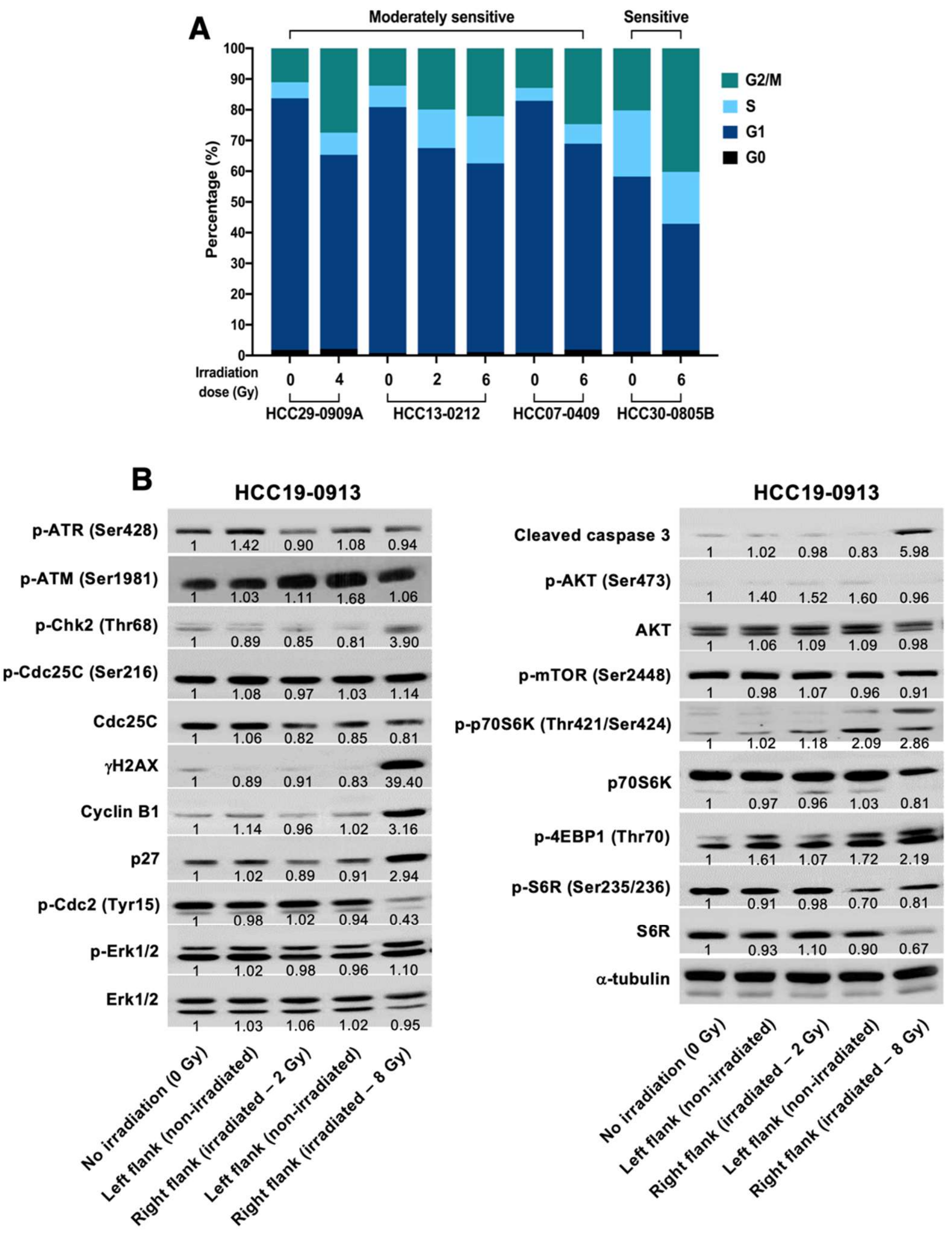

Figure S1. The effects of radiotherapy on cell cycle in vitro and the effects of localized radiotherapy on the DNA repair pathway, receptor tyrosine kinases (RTKs), and its downstream targets in the HCC19-0913 model. Radiotherapy (RT) caused G2M cell cycle arrest across all cell lines (**A**). HCC19-

0913 tumors were implanted, irradiated, and collected 2 days after RT as previously described. Tumor tissues were collected on day 2 post-RT, and lysates were subjected to Western blot analysis and quantification analysis as described in Appendix A. The intensity ratio of each band is expressed as the fold change. Representative blots are shown. 8 Gy RT increased the expression of proteins involved in DNA repair, cell cycle, and apoptosis. Representative blots are shown (**B**).

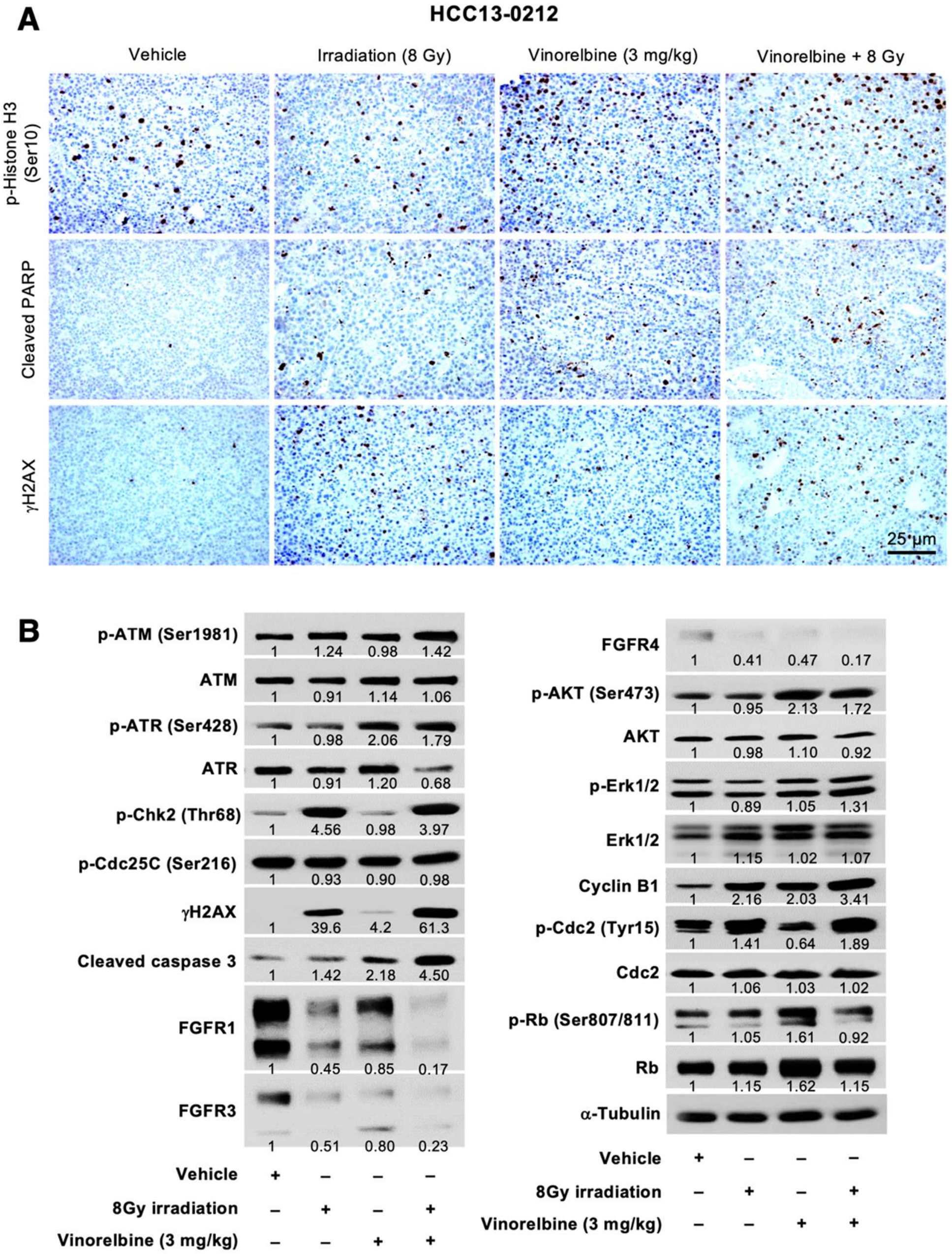

Figure S2. Effects of localized RT, Vinorelbine, and localized RT plus Vinorelbine on the HCC13-0212 PDX model. Two mice per group were subcutaneously implanted with HCC13-0212 tumors on both Figure 8. Gy on the right flanks. Treatments were initiated when the tumor sizes reached approximately 500–650 mm^3. Twelve hours after RT, control non-irradiated and irradiated mice were treated with 3 mg/kg Vinorelbine. Tumor tissues were collected on day 2 after RT, fixed, and then processed for immunohistochemistry as described in Appendix A. Sections from the vehicle-, RT-, Vinorelbine-, and RT/Vinorelbine-treated tumors were stained for p-Histone H3 Ser10, cleaved PARP, and γH2AX (**A**). Images were taken on an Olympus BX60 microscope (Olympus, Japan). Bars: 25 μm.

The same tumor tissues were subjected to Western blot and quantification analyses as described in Appendix A. The intensity ratio of each band is expressed as the fold change. Representative blots are shown (**B**).

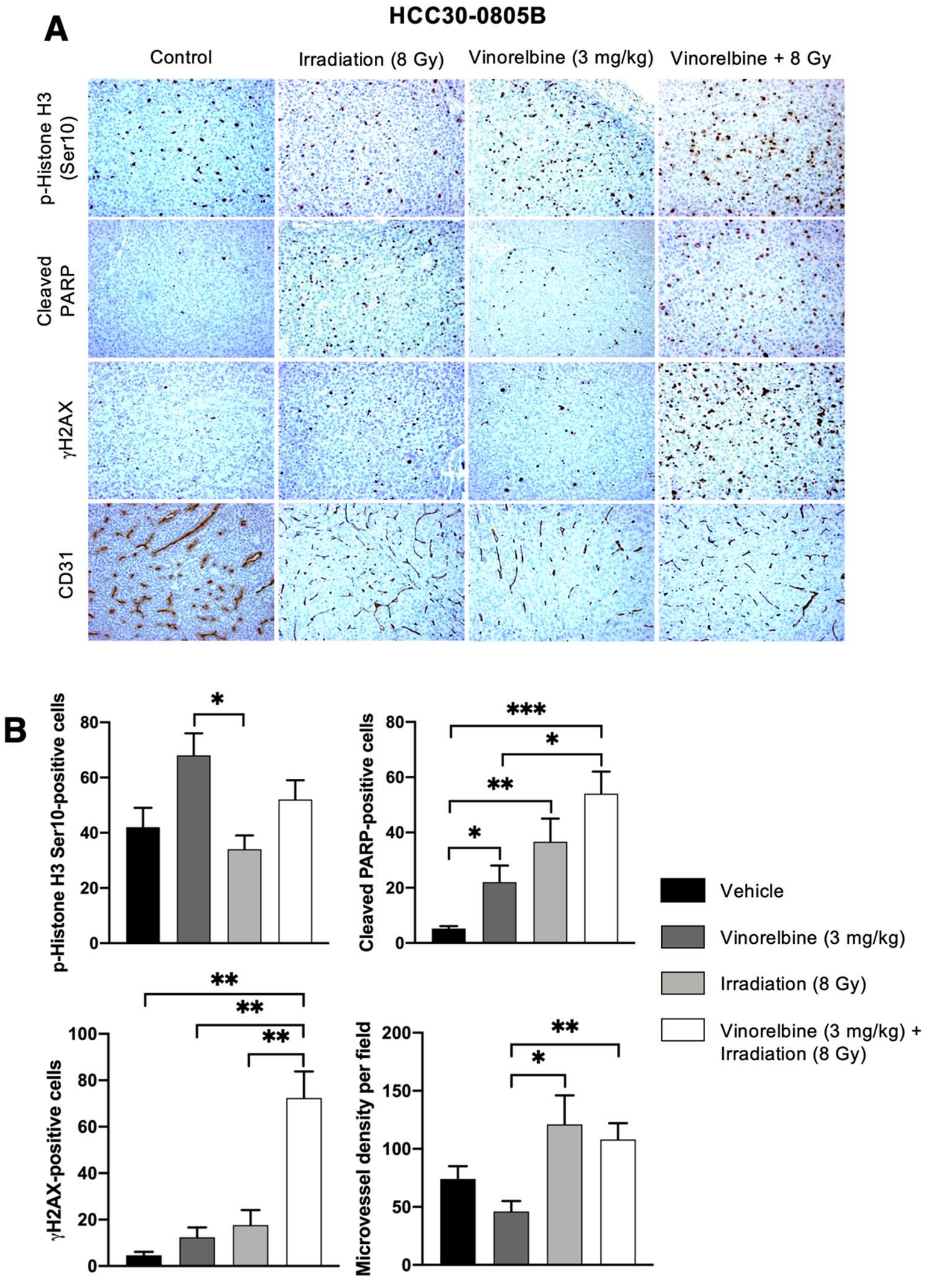

FigureS 3. Effects of localized RT, Vinorelbine, and localized RT plus Vinorelbine on the HCC30-0805B PDX model. Mice were subcutaneously implanted with HCC30-0805B tumors and subsequently irradiated with 8 Gy. Treatments were initiated when the tumor sizes reached approximately 500–650 mm^3. Twelve hours after RT, control non-irradiated and irradiated mice were treated with 3 mg/kg Vinorelbine. Tumor tissues were collected on day 2 after RT, fixed, and then processed for immunohistochemistry as described in Appendix A. Sections from the vehicle-, RT-, Vinorelbine-, and RT/Vinorelbine-treated tumors were stained for p-Histone H3 Ser10, cleaved PARP, γH2AX, and CD31 (**A**). Images were taken on an Olympus BX60 microscope (Olympus, Japan). Bars: 25 μm. The number of staining positive cells was quantified and is expressed as the number of positive cells per 1000 cells as described in Appendix A (**D**). * $p < 0.05$; ** $p < 0.01$; *** $p < 0.001$, Student's t-test (**B**).

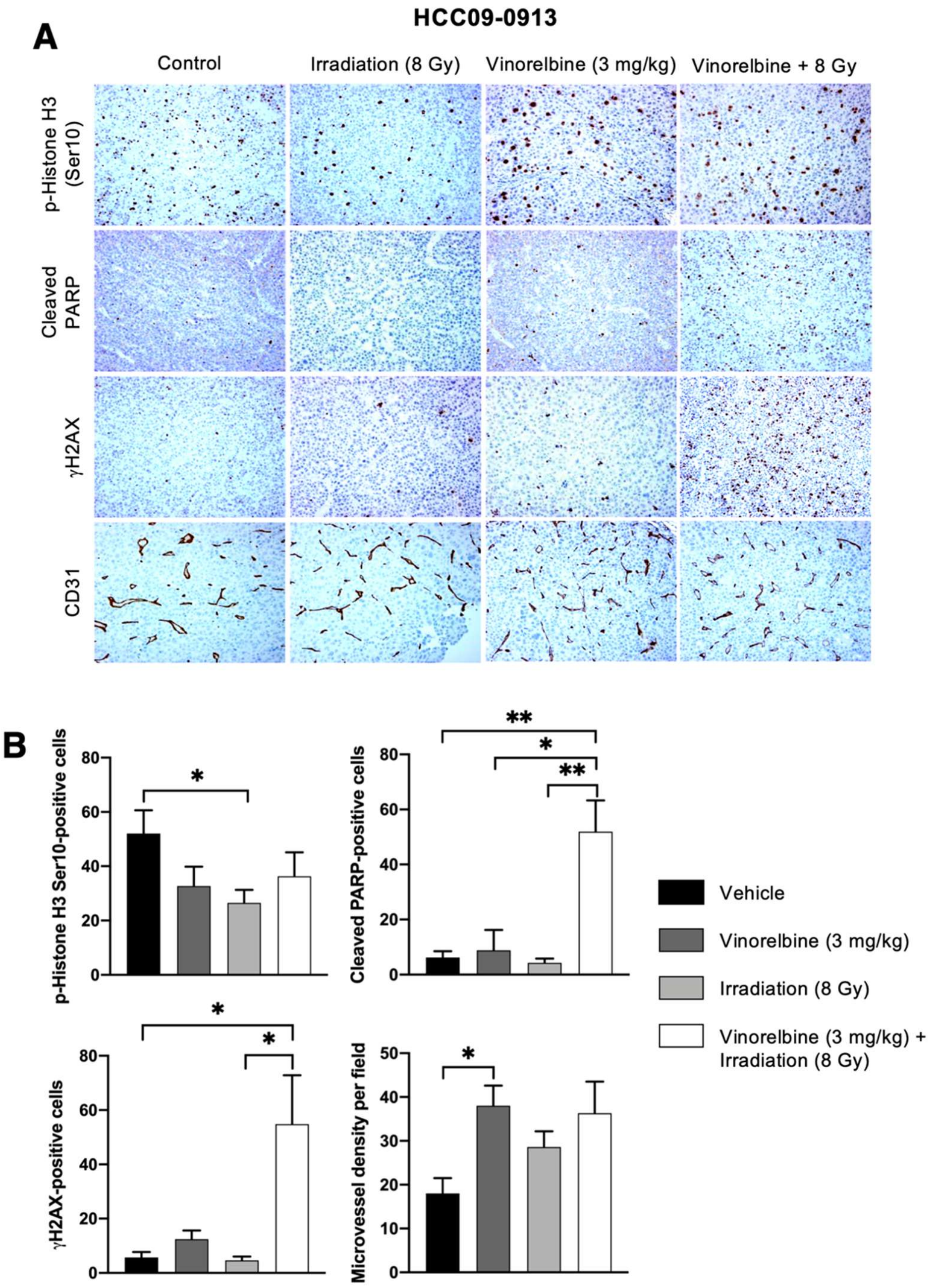

Figure S4. Effects of localized RT, Vinorelbine, and localized RT plus Vinorelbine on the HCC09-0913 PDX model. Mice were subcutaneously implanted with HCC09-0913 tumors and subsequently irradiated with 8 Gy. Treatments were initiated when the tumor sizes reached approximately 500–650 mm^3. Twelve hours after RT, control non-irradiated and irradiated mice were treated with 3 mg/kg Vinorelbine. Tumor tissues were collected on day 2 after RT, fixed, and then processed for immunohistochemistry as previously described. Sections from the vehicle-, RT-, Vinorelbine-, and RT/Vinorelbine-treated tumors were stained for p-Histone H3 Ser10, cleaved PARP, γH2AX, and CD31 (**A**). Images were taken on an Olympus BX60 microscope (Olympus, Japan). Bars: 25 μm. The number of staining positive cells was quantified and is expressed as the number of positive cells per 1000 cells as described in Appendix A. * $p < 0.05$; ** $p < 0.01$, Student's *t*-test (**B**).

A

Lines	Treatments	Tumour burden
HCC19-0913	0 Gy	100
	2 Gy	14 ± 4.6
	8 Gy	84.3 ± 1.7
	20 Gy	85.1 ± 4.3
	Vinorelbine (2 mg/kg)	62 ± 6.3
	Vinorelbine (3 mg/kg)	51 ± 5
	8Gy + Vinorelbine (2 mg/kg)	16 ± 4
	8Gy + Vinorelbine (3 mg/kg)	10 ± 3

B

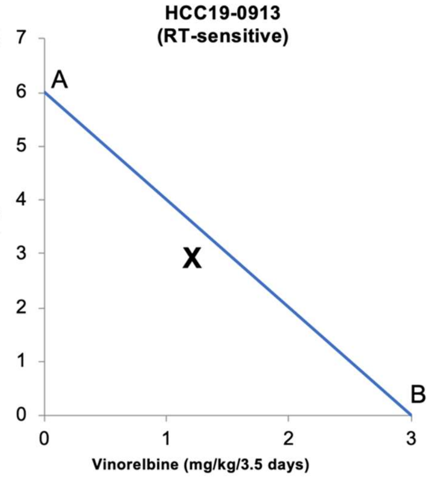

Figure S5. RT (8 Gy) plus Vinorelbine was synergistic. A graph of equally effective dose pairs for a single effect level (isobologram) was constructed using the median effect dose for 8 Gy, Vinorelbine, and 8 Gy plus Vinorelbine on the tumor, as described by Tallarida et al. [42]. Point A represents the dosage of RT alone (estimated to be approximately 6 Gy) to achieve approximately 50% tumor growth inhibition, while point B symbolizes the required Vinorelbine dosage alone (3 mg/kg every 3.5 days) to obtain 50% tumor growth inhibition. Any two dose combinations that fall on or close to the straight line that link point A to B are defined as additive. Any dose combination that falls to the left of the straight line is defined as superadditive/synergistic. Note that the dose pair such as point **X** in RT-sensitive HCC19-0913 that falls on the left side of the straight line is categorized as synergistic.
